# Recent advances in early screening and diagnosis of sepsis: a narrative review

**DOI:** 10.3389/fcimb.2026.1864446

**Published:** 2026-07-01

**Authors:** Xiao-Lian Zhou, Xiao-Bo Hu, Lu-Ying Shu, Yong-Sheng Wang

**Affiliations:** 1 Department of Pulmonary and Critical Care Medicine, West China School of Medicine, Sichuan University, Sichuan University Affiliated Chengdu Second People’s Hospital, Chengdu Second People’s Hospital, Chengdu, Sichuan, China; 2 Department of Respiratory and Critical Care Medicine, Chongzhou People’s Hospital, Chengdu, Sichuan, China

**Keywords:** biomarkers, early diagnosis, early screening, intelligent diagnosis, research progress, sepsis

## Abstract

Sepsis is a life-threatening organ dysfunction caused by a dysregulated host response to infection. Characterized by acute onset, rapid progression and high mortality, it poses a major global public health challenge in critical care medicine. Early accurate screening and diagnosis are crucial to improving patient prognosis and reducing mortality. In recent years, with the rapid advancement of medical technology, early screening and diagnostic strategies for sepsis have been continuously updated. Diagnostic approaches have evolved from traditional clinical scoring systems such as the The Sequential Organ Failure Assessment (SOFA) score and routine inflammatory biomarker detection to novel biomarkers including soluble Triggering Receptor Expressed on Myeloid cells-1(sTREM-1) and soluble cluster of differentiation 14 subtype (presepsin), as well as intelligent diagnostic techniques represented by Sepsis ImmunoScore and machine learning algorithms. Based on recent domestic and international research evidence, this narrative review summarizes the latest advances in early screening and diagnosis of sepsis, analyzes the strengths and limitations of various modalities, and provides references for early clinical identification and precise diagnosis of sepsis.

## 1 Introduction

Sepsis is a prevalent and intractable critical illness in intensive care units (ICU). Essentially, it refers to uncontrolled and imbalanced systemic inflammatory and immune responses triggered by infection. Revised by the Sepsis-3.0 consensus, it is defined as life-threatening organ dysfunction caused by dysregulated host responses to infection, highlighting the core pathological mechanisms of immune disorder and organ injury (Singer et al., 2016). According to the Global Burden of Disease Study, approximately 49 million sepsis cases occurred globally in 2017, leading to 11 million deaths and accounting for nearly 20% of all-cause mortality worldwide. Some 85% of cases and fat out-of-controlalities were recorded in low- and middle-income countries (Rudd et al., 2020). In China, sepsis patients make up over 20% of ICU admissions, with a 90-day mortality rate of 35.5%. Advanced age, high Sequential Organ Failure Assessment scores, heart failure, immunosuppression, elevated lactate levels and infection sites are all associated with 90-day mortality (Xie et al., 2020). Prognosis of sepsis is closely linked to intervention timing. Studies reveal that each one-hour delay in standard treatment raises mortality by 7.6%. For adult patients with septic shock, administration of effective antibiotics within one hour after hypotension identification can improve in-hospital survival rates (Kumar et al., 2006). Early screening, prompt diagnosis and initiation of targeted therapy are essential to reduce sepsis mortality and optimize patient outcomes. In recent years, the integrated application of molecular biology, artificial intelligence, imaging and other technologies has achieved remarkable breakthroughs in the early screening and diagnosis of sepsis, providing new insights and approaches for clinical practice. This paper conducts a narrative review and summarizes the latest relevant research progress.

## 2 Latest research on early screening of sepsis

### 2.1 Optimization and improvement of traditional clinical scoring systems

Sepsis diagnosis still centers on clinical judgment, and the disease cannot be confirmed or ruled out solely by a single biomarker, clinical scoring system or diagnostic test. Clinical scoring systems serve as classic tools for early sepsis screening. The most commonly used clinical assessment tools currently include the Systemic Inflammatory Response Syndrome (SIRS) criteria, quick Sequential Organ Failure Assessment (qSOFA) and National Early Warning Score (NEWS). Given its simple operation and independence from laboratory tests, qSOFA is widely applied for early sepsis screening in emergency departments and general wards. However, qSOFA should not be adopted as the primary standalone screening tool due to insufficient sensitivity and potential missed diagnosis of patients in the early deterioration stage. Evidence indicates that qSOFA presents low sensitivity and moderate specificity in predicting in-hospital mortality, 1-month mortality and ICU admission rate, suggesting its limited efficacy in patient screening and triage escalation (Lo et al., 2019). In a prospective observational study, Boekhoud et al. demonstrated that NEWS achieves superior discriminative accuracy over qSOFA in predicting in-hospital sepsis mortality, and patients receiving daily glucocorticoid doses exceeding 15 mg face particularly high in-hospital mortality risks (Boekhoud et al., 2024). Furthermore, researchers have established the lactate-modified qSOFA (LqSOFA) by combining qSOFA scores with lactate levels, which yields clinically significant sensitivity (69%) and specificity (79%) and markedly reduces the rate of missed diagnoses (Moncada-Gutiérrez et al., 2025). SOFA score is primarily used to evaluate the severity of organ dysfunction. Conventional SOFA relies heavily on ICU monitoring parameters, leading to monitoring gaps in general wards. The newly developed dynamic network nomogram model integrates clinical comorbidities such as hypertension and renal insufficiency as well as biomarkers including interleukin-6 (IL-6) and D-dimer, overcoming the application limitations of traditional SOFA. It has a negative predictive value of 0.832 and a positive predictive value of 0.511, making it suitable for early sepsis screening in non-ICU settings (Chen et al., 2025). In addition, composite scoring systems such as National Early Warning Score 2 (NEWS2) and Modified Early Warning Score (MEWS) incorporate physiological indicators including heart rate, respiratory rate, body temperature, blood pressure and mental status. A large cohort study involving over 221, 000 patients conducted by Piedmont S et al. verified that NEWS2 possesses the optimal overall diagnostic performance compared with MEWS, SIRS criteria and qSOFA, with a sensitivity of 73.1%, a specificity of 81.6% and an area under the receiver operating characteristic curve of 0.77 (Piedmont et al., 2024). These scoring systems are designed to identify high-risk patients with clinical deterioration caused by various etiologies, thus holding great auxiliary value in sepsis screening. The commonly used scoring tools for early sepsis screening are summarized in **Table 1** (Tavaré and O'Flynn, 2017; Inada-Kim, 2022; Gildea et al., 2024).

**Table 1:** Common risk stratification assessment tools for early screening of sepsis.

Assessment tool	Core variables	Main application	Applicable scenario	Scoring threshold and risk stratification
SOFA Score	Respiration, coagulation, liver function, circulation, nervous system, renal function	Quantify the severity of multiple organ failure and assess critical illness severity	All patients with sepsis	0–1 points: Low risk; 2–5 points: Moderate risk; ≥6 points: High risk
qSOFA Score	Respiration, consciousness, systolic blood pressure	Rapid sepsis screening and adverse prognosis prediction	Early screening in emergency department and hospitalized patients	0–1 points: Low risk; ≥ 2 points: High risk
NEWS2 Score	Respiration, oxygen saturation, oxygen demand, body temperature, systolic blood pressure, heart rate, consciousness	Condition monitoring; evaluate risks of death and intubation	Early screening in emergency and hospitalized settings	0–4 points: Low risk; 5–6 points/single item = 3 points: Moderate risk; ≥7 points: High risk
MEWS Score	Systolic blood pressure, heart rate, respiration, body temperature, consciousness	Early warning of disease deterioration and rapid identification of high-risk patients	Rapid assessment in pre-hospital, emergency department and general ward	0–4 points: Low risk; 5–6 points: Moderate risk; ≥7 points: High risk
SIRS score	Body temperature, heart rate, respiration, peripheral blood leukocytes	Screen systemic inflammatory response	All patients with sepsis	0–1 points: Low risk; 2–3 points: Moderate risk; ≥4 points: High risk

### 2.2 Research and application of novel screening tools

Routine laboratory indicators serve as important auxiliary methods for early screening of sepsis, which mainly reflect the systemic inflammatory response status by detecting inflammation-related markers in blood. With the advancement of portable detection technologies, novel rapid screening tools have been gradually applied in clinical practice. Portable lactate analyzers are widely adopted screening tools in recent years, applicable to specific scenarios including intensive care units, primary medical institutions and emergency triage. Raa et al. verified the clinical application of capillary lactate measurement devices in critically ill patients via a prospective study. The handheld Lactate Pro 2 analyzer showed good consistency with the reference method using arterial blood detection, and earlobe sites were more suitable for sampling than fingertips in critically ill patients (Raa et al., 2020). The research team led by Thongkhao developed a lactate biosensor for the determination of plasma lactate in human bodies. This biosensor achieves a rapid response time of 5 seconds, high selectivity and excellent long-term storage stability within 10 weeks, making it an ideal tool to promote the clinical application of lactate in the early diagnosis of sepsis in primary medical institutions (Thongkhao et al., 2024). Studies have demonstrated that point-of-care testing (POCT) for lactate used in busy emergency departments can effectively shorten the turnaround time of lactate results, improve compliance with 1-hour bundle therapy and optimize patient prognosis (Lee et al., 2024). Nevertheless, lactate presents low specificity for sepsis diagnosis. A clinical emergency sepsis screening scoring system combining NEWS with multiple biomarkers including lactate, CRP, PCT and NLR has been established, which exhibits favorable diagnostic efficiency with a sensitivity of 83.9% and a specificity of 72.2%, and can assist clinicians in identifying sepsis patients in emergency settings (Chen ZG. et al., 2023). In terms of cellular characteristic analysis, indicators such as monocyte distribution width (MDW) and neutrophil deformability (IntelliSep) have become research hotspots. Relevant reviews have confirmed that MDW is an effective and convenient diagnostic biomarker for sepsis with diagnostic efficacy comparable to PCT and CRP and high sensitivity, which can be well applied for early screening of mild sepsis patients in general wards and ICUs (Huang et al., 2023). A prospective cohort study indicated that IntelliSep can detect sepsis-related cellular biomechanical alterations by assessing neutrophil deformability. It acts as a rapid auxiliary diagnostic tool (within 10 minutes) for risk stratification of adverse clinical outcomes, and is suitable for emergency patients with infectious manifestations or suspected sepsis (O'Neal et al., 2024). In imaging screening, emergency ultrasound is particularly suitable for the screening of urosepsis due to its advantages of convenient operation, radiation-free property and bedside availability. Relevant studies have confirmed that emergency ultrasound has equivalent diagnostic efficacy to CT scanning for urosepsis, and is more preferable for rapid emergency screening (Dragoescu et al., 2024).

### 2.3 Precise targeting and screening of high-risk populations

Sepsis shows distinct high-risk population characteristics, predominantly involving neonates, elderly individuals and patients with underlying tumor diseases. Such populations are prone to developing sepsis after infection and often present with atypical early symptoms. In recent years, studies have focused on precise screening strategies for high-risk groups to improve the efficiency of early identification by establishing targeted screening protocols. A retrospective study demonstrated that combined detection of methylthioadenosine and heparin-binding protein facilitates the early diagnosis of neonatal sepsis, and sustained elevation of both indicators indicates a high risk of mortality in septic neonates (Zheng et al., 2020). Given the declined physiological function, impaired immunity and atypical clinical manifestations in elderly patients, a retrospective study constructed an early sepsis screening model based on non-invasive physiological parameters, incorporating non-specific indicators including mental status, body temperature, blood pressure and urine output. The area under the curve (AUC) of this regression model for diagnosing sepsis in elderly patients was 0.671, which was superior to 0.572 of the SIRS score and 0.631 of the qSOFA score (Liu et al., 2024). Moreover, for immunocompromised populations, especially patients with hematological malignancies and solid tumors complicated with sepsis, inflammatory markers such as IL-6, PCT and CRP serve as effective early diagnostic indicators. Combined biomarker panels including IL-6+PCT and LYM+NEU achieved an AUC of 0.866, exhibiting favorable diagnostic efficacy. Among biomarker combinations for patients with solid tumors, CRP+PCT was the most efficient combination with an AUC of 0.658 (Carcò et al., 2025).

## 3 Latest research progress in the early diagnosis of sepsis

### 3.1 Research progress of biomarkers in the early diagnosis of sepsis

Biomarkers are detectable indicators that reflect the pathophysiological status of the organism. They can rapidly and objectively indicate the onset and progression of sepsis, serving as vital tools for the early diagnosis of sepsis. At present, research on sepsis-related biomarkers has expanded from traditional inflammatory indicators to multi-dimensional domains such as immune-related molecules and metabolic biomarkers, with newly discovered biomarkers achieving markedly improved specificity and sensitivity.

#### 3.1.1 Optimized application of traditional biomarkers

Blood culture is the gold standard for etiological diagnosis, yet it has a low positive detection rate. A negative blood culture result cannot rule out sepsis. Even if pathogens are detected positively, sepsis cannot be directly confirmed in the absence of consistent host responses and manifestations of organ dysfunction. Factors affecting the detection rate of blood culture include the pre-test probability of bacteremia, application of anaerobic culture bottles, and prior antibiotic exposure. A retrospective clinical study enrolling more than 5, 000 patients conducted by Anna et al. demonstrated that switching the sampling strategy from the mainstream multi-site sampling (MSS) to single-site sampling (SSS) achieves a higher positive rate, larger valid sample volume and fewer single blood culture sets. The advantages of SSS should be taken into account in future sepsis guidelines (Ekwall-Larson et al., 2022). Vashti et al. also indicated that single-site sampling can elevate pathogen detection rate and true positive rate, and exhibits superior performance in identifying bacteremia compared with multi-site sampling. Collecting more than four culture bottles yields no additional benefits for bacterial detection, and single-site sampling avoids the increased contamination risk caused by repeated venipuncture (Vashti et al., 2025).

A meta-analysis involving over 2, 000 adult patients with severe sepsis and septic shock revealed that serum PCT levels are closely correlated with the severity of bacterial infection, which can differentiate bacterial from viral infections and guide antibiotic therapy (Zaki et al., 2024). The normal PCT level in healthy populations is less than 0.15 ng/mL, while it is generally higher than 2.0 ng/mL in patients with sepsis. Nevertheless, PCT shows no marked elevation in sepsis induced by viral or fungal infections. As stated in a review by Cortegiani et al., patients with candidemia present significantly lower PCT levels than those with bacteremia (Cortegiani et al., 2019).

**Figure 1:**
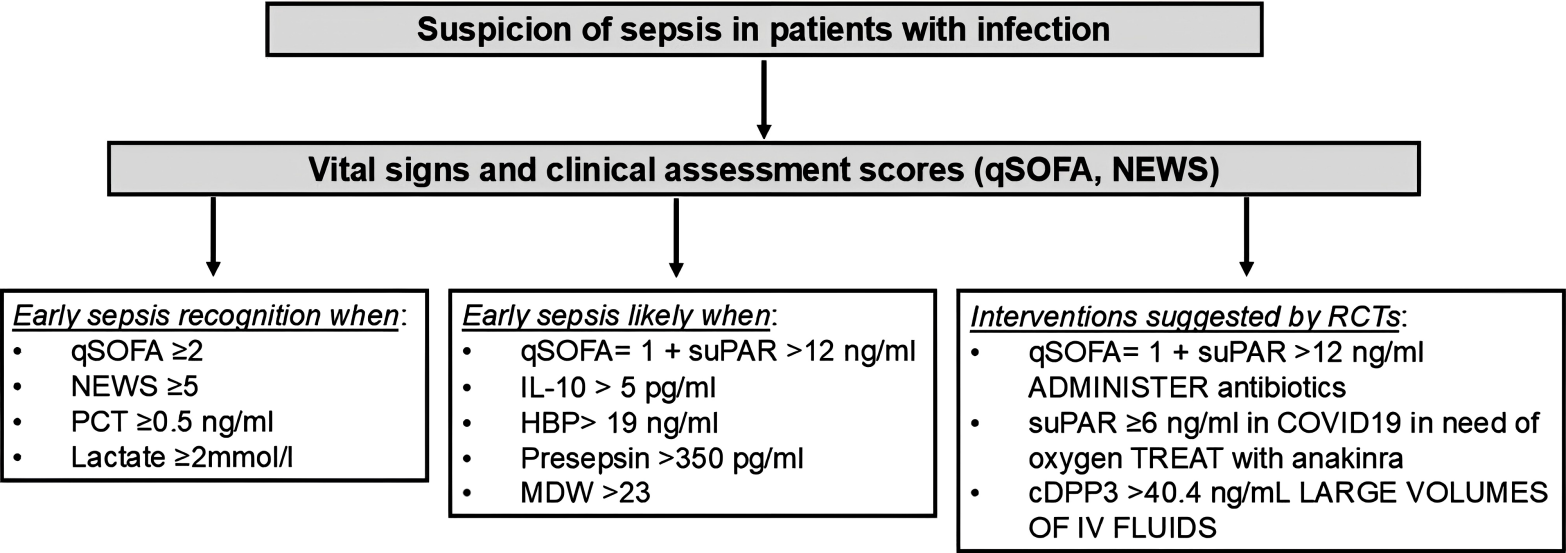
Proposed algorithm for early recognition and management of sepsis based on biomarkers including heparin-binding protein (HBP), monocyte distribution width (MDW), interleukin-10 (IL-10), prostaglandin, procalcitonin (PCT), and C-reactive protein (CRP).

As an early inflammatory cytokine, IL-6 peaks within 2 hours after inflammatory activation and responds to infection more rapidly than CRP and PCT, but it lacks sufficient specificity (He et al., 2024). A prospective observational study recruiting more than 400 adult patients from 2016 to 2017 compared the ability of clinicians and IL-6 in identifying infection. The AUC of clinical judgment for distinguishing infectious status was 0.78 with a 95% confidence interval of 0.74–0.82. The combined application of IL-6 detection and clinical judgment increased the AUC to 0.84 (95%CI: 0.79–0.87) (Henning et al., 2020). **Table 2** summarizes the advantages and disadvantages of the above traditional diagnostic methods. Among diverse biomarkers for early sepsis identification, evidence-based biomarkers capable of predicting the progression from suspected infection to overt sepsis are conducive to timely intervention. Members of the Bourika research team proposed an algorithm for early sepsis recognition and management recommendations, as shown in **Figure 1** (Bourika et al., 2025).

**Table 2:** Comparison of traditional diagnostic methods for sepsis.

Traditional diagnostic methods	Advantages	Limitations	Application scope
Blood culture	The gold standard for etiological diagnosis	Low positive rate, affected by the number of blood sample sets, blood collection volume, application of anaerobic culture bottles and prior antibiotic medication history	Patients with bloodstream infection, severe ICU infection and septic shock
PCT	High specificity; capable of evaluating disease severity; guiding the rational use of antibiotics	Influenced by drugs; unable to identify specific pathogens; insufficient diagnostic sensitivity in the ultra-early stage of infection and immunocompromised populations	Sepsis caused by bacterial infections in adults and children; superior diagnostic value for sepsis secondary to community-acquired infections
IL-6	High sensitivity, able to assess disease severity and wide application range	Poor specificity, unable to guide antibiotic administration, less stable prognostic evaluation effect than PCT	Rapid emergency preliminary screening, perioperative infection early warning and dynamic monitoring of critically ill patients

#### 3.1.2 Exploration and application of novel biomarkers

Sepsis is a heterogeneous clinical syndrome characterized by dysregulated host responses, accompanied by multiple pathological alterations including organ dysfunction, immune paralysis, metabolic reprogramming, endothelial dysfunction and abnormal coagulation function. With the advancement of molecular biological techniques, a variety of novel biomarkers have been identified, mainly involving immune-related molecules, cellular metabolic markers and microRNAs, which provide new targets for the early diagnosis of sepsis.

Immune-related molecules have become a research hotspot in recent years. Immune dysregulation constitutes the core pathological mechanism of sepsis, featured by impaired immune cell function and aberrant expression of immune molecules. sTREM-1 and presepsin are well-established immune-related biomarkers. Primarily expressed on the surface of neutrophils and monocytes, sTREM-1 is released into the bloodstream in its soluble form after infection, which is markedly elevated in septic patients and positively correlated with disease severity. A meta-analysis confirmed that its area under the summary receiver operating characteristic curve (AuROC) for sepsis diagnosis was 0.88, with a pooled sensitivity of 0.82 and specificity of 0.81, enabling effective differentiation between sepsis and non-infectious inflammation (Chang et al., 2020). Presepsin rises rapidly earlier than PCT and CRP following bacterial infection, making it particularly suitable for early sepsis diagnosis. It achieves a diagnostic sensitivity of 89.1% and the highest specificity of 88.9%, and maintains favorable diagnostic efficacy in immunocompromised patients. Nevertheless, clinicians should note that renal insufficiency may cause its false elevation (Baxla et al., 2025).

**Figure 2:**
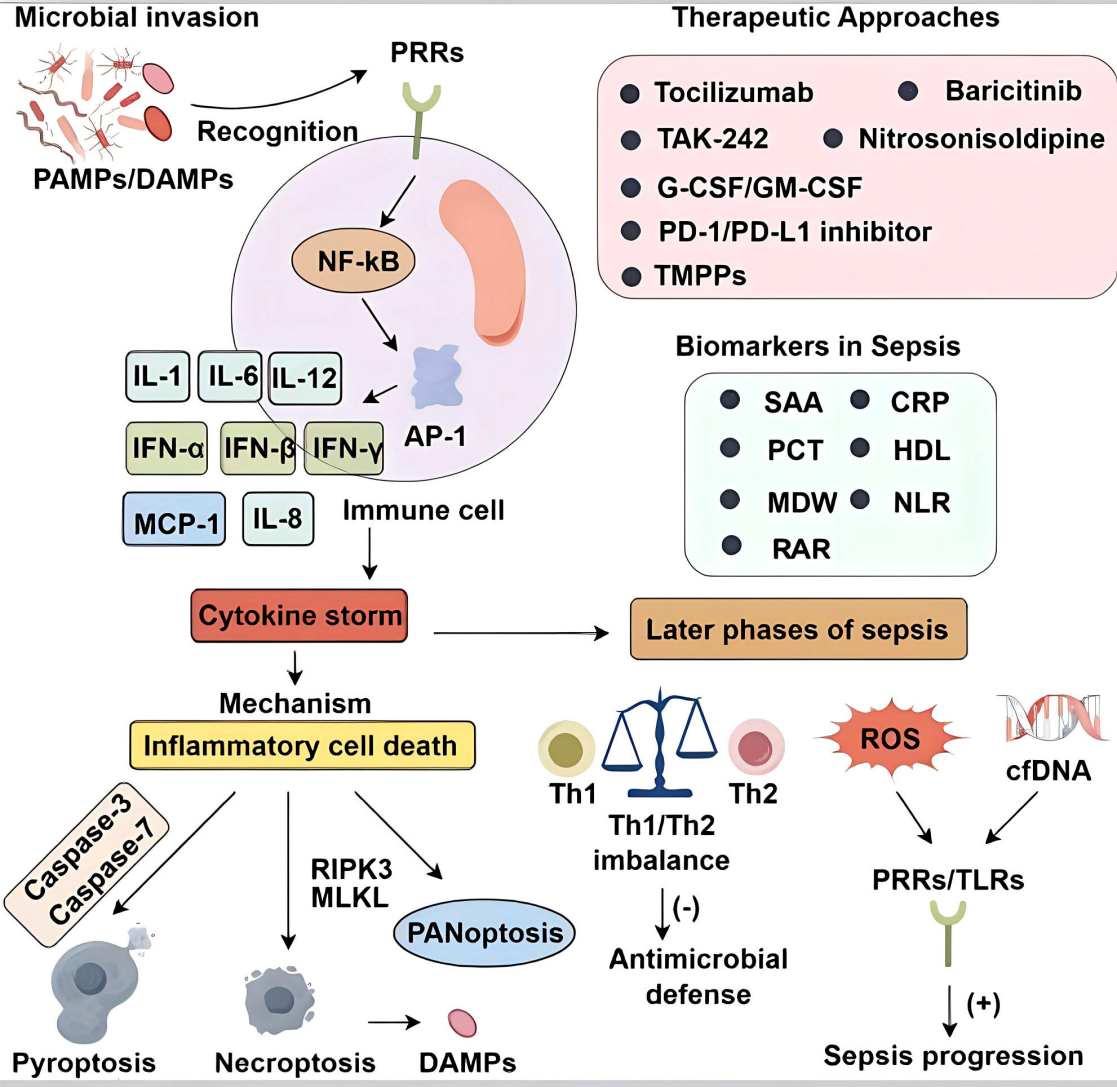
Application of cytokines in sepsis.

Cytokines and immune cell biomarkers have also attracted extensive research attention. Recently emerging biomarkers such as serum amyloid A (SAA), high-density lipoprotein (HDL), red cell distribution width to albumin ratio (RAR), neutrophil-to-lymphocyte ratio (NLR) with a cut-off value ranging from 3 to 5, and monocyte distribution width (MDW) with an optimal diagnostic threshold of 23.5, exhibit diagnostic performance comparable to CRP and PCT, revealing promising diagnostic and prognostic values. The clinical application of cytokines in sepsis is illustrated in **Figure 2** (You, 2025). Cytokines including IL-6, IL-8 and tumor necrosis factor-α (TNF-α) are significantly increased in the peripheral blood of septic patients, and their levels are closely associated with disease severity and clinical prognosis. A prospective study conducted by Zhang et al. verified that IL-8 possesses strong prognostic value for mortality in elderly septic patients, and the combination of IL-8 and SOFA score can improve the predictive efficacy of mortality risk in this population (Zhang et al., 2024). Distinct alterations are observed in the proportions of peripheral blood CD4^+^ T cells, B cells and their subsets in septic patients. The occurrence of immune cell dysfunction and immune exhaustion phenotypes is linked to immunosuppressive status and increased mortality (Martin et al., 2020). Basophil depletion can serve as an early diagnostic clue for sepsis. Relevant studies have demonstrated that basophil deficiency is positively correlated with immunosuppression, indicating that basophil-mediated immunity has potential value in predicting 28-day mortality among ICU patients (Chen X. et al., 2023).

MicroRNAs (miRNAs) are a class of small non-coding RNAs derived from both immune and non-immune cells, playing pivotal roles in immune imbalance, inflammatory storm and organ injury during sepsis progression (Xie et al., 2025). Xiao et al. explored the potential of exosome-derived miRNAs as biomarkers for early detection and prognostic evaluation of sepsis, which may lay a foundation for targeted regulation of septic responses and individualized therapeutic strategies (Xiao et al., 2024). Numerous recent studies have confirmed abnormal expression levels of miR-146a, miR-21 and miR-155 in the blood of septic patients. Among them, miR-146a can inhibit inflammatory responses via regulating the TLR4 signaling pathway, while miR-23b is proposed as a biomarker reflecting disease severity (Valsamaki et al., 2024). A meta-analysis performed by Zheng et al. showed that the overall diagnostic performance of miRNAs presented a pooled sensitivity of 0.76 and pooled specificity of 0.77. Subgroup analysis revealed that miR-155-5p yielded the highest SROC area under the curve (AUC = 0.85), with a pooled sensitivity of 0.71 and pooled specificity of 0.82 (Zheng et al., 2023). **Table 3** summarizes the diagnostic differences of the above novel biomarkers for sepsis.

**Table 3:** Comparison of novel biomarkers in the diagnosis of sepsis.

Novel biomarkers	Advantages	Limitations	Application scope
sTREM-1	Strong specificity and high sensitivity; capable of evaluating disease severity and clinical prognosis.	No obvious elevation in ultra-early sepsis; also increased in fungal infection; high detection cost and low popularization in primary medical institutions.	Suitable for patients with suspected severe sepsis, differentiating inflammatory types and judging disease severity.
presepsin	Rises rapidly; not affected by trauma and surgical stress; available for early risk stratification.	Inferior specificity to sTREM-1; false elevation may occur in renal insufficiency and chronic inflammation; relatively high detection cost.	Ultra-early screening in emergency department
IL-8	High sensitivity; decreases rapidly after treatment, conducive to dynamic monitoring.	Poor specificity; short half-life and easily affected by blood collection time.	Mostly used in combined detection to assist in evaluating the degree of inflammatory activation.
RAR	Convenient detection; able to assess disease severity and prognosis.	Low specificity, easily interfered by liver diseases, anemia and other disorders.	Only used as an auxiliary indicator for combined evaluation and prognostic judgment.
Basophil	Early decrease in cell count; simple detection with low cost; available for dynamic observation of disease recovery.	Extremely low specificity; susceptible to interference by drugs and underlying diseases.	Used for auxiliary judgment via routine blood tests, not applied for independent diagnosis.
miRNA	High stability and resistance to degradation; great potential for molecular diagnosis; high diagnostic accuracy.	No unified diagnostic cut-off value; complicated detection procedures and high cost; low clinical popularization.	Basic research and mechanism exploration, not suitable for routine clinical screening at present.

### 3.2 Innovation and application of diagnostic technologies

#### 3.2.1 Advances in imaging diagnostic technologies

Imaging techniques are mainly applied to identify infection sites and assess organ dysfunction, providing radiological evidence for the diagnosis of sepsis. In recent years, the optimization of imaging technologies has been predominantly focused on the integration of functional imaging and artificial intelligence. Contrast-enhanced ultrasound (CEUS) can evaluate tissue perfusion and reflect early tissue hypoxia and organ damage, thus holding great value in the early diagnosis of sepsis-associated acute kidney injury (SA-AKI). Researchers established a deep learning ultrasound radiomics model (DLUR) based on destruction-replenishment contrast-enhanced ultrasound (DR-CEUS). The model achieved an AUC of 0.921 in predicting SA-AKI, which was significantly superior to visual assessment by senior ultrasonologists with an AUC of 0.829, enabling early identification of sepsis-induced organ dysfunction (Yu et al., 2025). In an experimental study on the early diagnosis of sepsis-associated encephalopathy (SAE) in mice, dynamic PET/CT and MRI were proven to possess early diagnostic potential. Moreover, MRI has higher soft tissue resolution than PET/CT and allows classification of more refined brain regions (Zhu et al., 2022).

#### 3.2.2 Advances in molecular biological diagnostic technologies

PCR serves as the fundamental technique for molecular diagnosis, including conventional PCR and real-time fluorescent quantitative PCR. The research by Favaro’s team evaluated two novel probe-based multiplex real-time PCR systems, namely SEPSI ID panel and SEPSI DR panel. They can rapidly identify bacterial and fungal pathogens as well as antibiotic resistance genes, covering 29 microbial species. Requiring no nucleic acid extraction, the test results can be obtained within approximately one hour (Favaro et al., 2025). A meta-analysis has confirmed that assays targeting bacterial 16S rRNA and viral nucleic acids feature short detection cycles and high sensitivity, enabling identification of pathogens at low concentrations, which makes them suitable for early diagnosis of sepsis (Drevinek et al., 2023). As an innovative PCR method, droplet digital PCR (ddPCR) allows absolute quantitative detection of pathogens. In the quantification of bacteria and fungi, ddPCR outperforms qPCR in repeatability, sensitivity and stability, and is capable of amplifying long-segment target genes, thus being especially applicable to the diagnosis of infections caused by trace pathogens (Wang et al., 2022).

Metagenomic next-generation sequencing (mNGS) has become a research hotspot in recent years. It can directly sequence all microbial nucleic acids in specimens without microbial culture and achieve rapid pathogen identification. A multicenter prospective study demonstrated that the positive coincidence rate of mNGS in specimens from infection sites reached 92.0%, which was remarkably higher than 51.1% of traditional microbial detection methods. It facilitates etiological diagnosis and guides individualized antibiotic therapy (Xu et al., 2025). Bronchoalveolar lavage fluid (BALF) is the most common specimen type for mNGS. This technique can detect rare pathogens that are hardly identified by traditional culture methods, and is more applicable to patients with complicated conditions, culture-negative results, immunodeficiency and refractory infections unresponsive to conventional treatment (He et al., 2022). Nevertheless, mNGS has inherent limitations including high testing costs, long turnaround time, high risk of exogenous specimen contamination, and difficulty in distinguishing colonizing bacteria from pathogenic bacteria.

Multi-omics technology has gained increasing research attention recently. By integrating multi-dimensional data such as host gene expression, protein synthesis and metabolite alterations, it can elucidate the pathophysiological mechanisms of sepsis and screen specific diagnostic biomarkers. Transcriptomics can screen differentially expressed genes in the early stage of sepsis and reflect the host immune status against infection (Taha et al., 2025). Proteomics research not only reveals the molecular characteristics of immunity disturbance, metabolic disorders and organ injury in sepsis, but also provides core targets for the development of precise diagnostic tools, monitoring indicators and targeted therapies. Metabolomics is able to detect changes in blood metabolites, which can act as potential biomarkers for early sepsis diagnosis (Jin et al., 2025).

#### 3.2.3 Application of artificial intelligence diagnostic technology

Artificial intelligence constructs early diagnostic models for sepsis by integrating clinical data, biomarker profiles and imaging data, enabling accurate and rapid diagnosis, which has become a popular research direction in recent years. Although specific mechanisms vary among different models, most analyze core indicators associated with sepsis progression, including vital signs, demographic characteristics, laboratory findings, comorbidities and clinical management data (Hassan et al., 2021). Islam MM et al. conducted a meta-analysis of observational studies adopting machine learning for sepsis prediction. The pooled area under the summary receiver operating characteristic curve reached 0.89 with a sensitivity of 0.81 and specificity of 0.72 when predicting sepsis 3 to 4 hours before onset. By comparison, the pooled SAUROC values of SIRS, MEWS and SOFA were 0.70, 0.50 and 0.78 respectively (Islam et al., 2019). Given the high incidence of sepsis among critically ill patients, Michael et al. reviewed and evaluated machine learning-based models for sepsis prediction in ICU settings, aiming to advance early sepsis recognition (Moor et al., 2021). Machine learning-derived predictive models serve as effective auxiliary tools. They can visually display variable weights at the individual patient level and quantify risk contributors, enhancing clinicians’ acceptance of model outputs and mitigating black-box drawbacks.

#### 3.2.4 Functional diagnostic techniques for host response

Reduced expression of monocyte human leukocyte antigen-DR (mHLA-DR) is correlated with immune paralysis, and its expression level directly reflects the antigen-presenting capacity of monocytes. Studies have demonstrated that low mHLA-DR expression is associated with increased nosocomial infection and mortality following sepsis (Landelle et al., 2010). Its expression is susceptible to interference from age and underlying diseases, and flow cytometry is the predominant method used to detect surface molecule expression on peripheral blood monocytes. Endotoxin tolerance is defined as diminished responsiveness to lipopolysaccharide (LPS) re-challenge after initial endotoxin exposure. Decreased TNF-α release upon *in vitro* LPS stimulation indicates endotoxin tolerance and innate immune dysfunction (Cavaillon and Adib-Conquy, 2006). Its level is affected by host immune status and pharmacological intervention. Both biomarkers can objectively evaluate immune cell responsiveness, determine the severity of immune disorder, and predict risks of secondary infection and adverse clinical outcomes. Current relevant studies are mostly retrospective observational analyses, and large-sample prospective clinical trials are required to verify their diagnostic efficacy.

### 3.3 Establishment of multi-dimensional integrated diagnostic models

The *2026 SSC Guidelines* propose a stratified diagnostic strategy, classifying infectious indicators into four grades: definite sepsis, probable sepsis, possible sepsis and unlikely sepsis (**Table 4**) (Prescott et al., 2026). Dynamic changes of multiple indicators including WBC, CRP and PCT, combined with quantitative assessment of organ dysfunction via SOFA score and lactic acid level, facilitate accurate diagnosis of sepsis. Elevated SOFA score, CRP, PCT and lactic acid are significant predictors of sepsis mortality (Daud et al., 2024). Bhargava et al. established the Sepsis ImmunoScore assay. This artificial intelligence tool integrates electronic health records, specific biomarkers and cytokine measurements. It achieves an AUC of 0.85 in the derivation cohort and 0.81 in external validation, and is an FDA-authorized AI/ML risk prediction model. The score enables 24-hour risk stratification for sepsis onset, with secondary endpoints including in-hospital mortality, ICU admission, mechanical ventilation and vasopressor administration (Bhargava et al., 2024). Sepsis ImmunoScore presents excellent performance in early risk identification and severity stratification. Nevertheless, it is restricted by population bias, temporal limitations, poor clinical workflow compatibility and alert fatigue, and can only serve as an auxiliary AI warning tool. Bacteremia can be detected via mNGS in half of sepsis patients. PCT, IL-6 and hsCRP help distinguish Gram-negative, Gram-positive and fungal bacteremia, providing evidence for targeted treatment (Zhou et al., 2025).

**Table 4:** Terminology of sepsis in the 2026 SSC guidelines.

Different terminologies for sepsis	Terminology definition
Definite sepsis	Sepsis is confirmed based on history, clinical examination, and diagnostic testing. An alternative diagnosis is very unlikely
Probable sepsis	High suspicion for sepsis. Sepsis is the most likely diagnosis based on history, clinical examination, and diagnostic testing. An alternative diagnosis is less likely
Possible sepsis	Moderate suspicion for sepsis. Sepsis is a possible diagnosis; however, an alternative diagnosis is also likely based on history, clinical examination, and diagnostic testing
Unlikely sepsis	Low suspicion for sepsis. Clinical assessment is not consistent with sepsis, or an alternate diagnosis is more likely based on history, clinical exami− nation, and diagnostic testing

## 4 Future development directions

In summary, sepsis possesses complex and diverse pathogenesis, with immune disorders, inflammatory imbalance and multiple organ injuries intertwined with each other. A single diagnostic indicator is no longer sufficient to meet clinical requirements for early identification, accurate classification and prognostic assessment of sepsis. With unique advantages, various novel immune biomarkers, cellular metabolic indicators and non-coding RNAs have gradually compensated for the shortcomings of traditional detection methods, offering new strategies for the precise diagnosis and treatment of sepsis. Future researches on sepsis will focus on establishing early warning systems based on artificial intelligence and big data, so as to achieve risk prediction among high-risk infected populations, precise stratification of sepsis and individualized targeted immune intervention. Such strategies are expected to further shorten the diagnostic window and optimize the prognosis of critically ill patients.

## 5 Conclusion

In recent years, traditional clinical scoring systems have been continuously optimized, and the application of routine laboratory indicators has become more standardized. The emergence of novel biomarkers, molecular diagnostic techniques and artificial intelligence-assisted diagnostic methods has remarkably improved the accuracy and timeliness of early screening and diagnosis for sepsis. In the future, with the research and development of new biomarkers, the optimization of molecular detection technologies and the in-depth application of artificial intelligence, the early screening and diagnosis of sepsis will develop toward precision, intelligence and convenience. It will provide stronger support for early detection, early diagnosis and early intervention of sepsis in clinical practice, ultimately improve patients’ clinical prognosis and reduce the disease burden.
